# Parent-son decision-making about human papillomavirus vaccination: a qualitative analysis

**DOI:** 10.1186/1471-2431-12-192

**Published:** 2012-12-14

**Authors:** Andreia B Alexander, Nathan W Stupiansky, Mary A Ott, Debby Herbenick, Michael Reece, Gregory D Zimet

**Affiliations:** 1 Center for Sexual Health Promotion, Indiana University, Bloomington, IN, USA; 2 Section of Adolescent Medicine, Indiana University, Indianapolis, IN, USA; 3 Indiana University School of Medicine, 410 West Tenth Street, HS 1001, Indianapolis, IN, 46202, USA

**Keywords:** Human papillomavirus vaccin, Adolescent males, Parents, Decision-making, Dyadic decision-making

## Abstract

**Background:**

Licensed for use in males in 2009, Human Papillomavirus (HPV) vaccination rates in adolescent males are extremely low. Literature on HPV vaccination focuses on females, adult males, or parents of adolescent males, without including adolescent males or the dynamics of the parent-son interaction that may influence vaccine decision-making. The purpose of this paper is to examine the decision-making process of parent-son dyads when deciding whether or not to get vaccinated against HPV.

**Methods:**

Twenty-one adolescent males (ages 13–17), with no previous HPV vaccination, and their parents/guardians were recruited from adolescent primary care clinics serving low to middle income families in a large Midwestern city. Dyad members participated in separate semi-structured interviews assessing the relative role of the parent and son in the decision regarding HPV vaccination. Interviews were recorded, transcribed, and coded using inductive content analysis.

**Results:**

Parents and sons focused on protection as a reason for vaccination; parents felt a need to protect their child, while sons wanted to protect their own health. Parents and sons commonly misinterpreted the information about the vaccine. Sons were concerned about an injection in the penis, while some parents and sons thought the vaccine would protect them against other sexually transmitted infections including Herpes, Gonorrhea, and HIV. Parents and sons recalled that the vaccine prevented genital warts rather than cancer. The vaccine decision-making process was rapid and dynamic, including an initial reaction to the recommendation for HPV vaccine, discussion between parent and son, and the final vaccine decision. Provider input was weighed in instances of initial disagreement. Many boys felt that this was the first health care decision that they had been involved in. Dyads which reported shared decision-making were more likely to openly communicate about sexual issues than those that agreed the son made the decision.

**Conclusion:**

Parents and sons play an active role in the decision-making process, with an individual’s role being influenced by many factors. The results of this study may be used to guide the messages presented by clinicians when recommending the HPV vaccine, and future vaccine uptake interventions.

## Background

Human papillomavirus (HPV) is the most common sexually transmitted infection (STI) in both men and women [1]. In 2006 the HPV vaccine was licensed in females ages 9-26 [2], and given a routine recommendation by the U.S. Advisory Committee on Immunization Practices (ACIP) [3]. This vaccine was actively promoted by pharmaceutical companies and quickly became a part of the health care agenda, resulting in moderate levels of vaccination. In 2009, the quadrivalent HPV vaccine was licensed in the U.S. for males ages 9–26 [4] as well, but with a permissive, rather than routine, recommendation by the ACIP [5], which left the decision to vaccinate to the discretion of the health care provider and/or parents. Additionally, this licensure and recommendation was not followed by a strong advertising campaign, or given a great deal of attention by the health care system. In 2010, 1.4% of males ages 13–17 received at least one dose of the HPV vaccine [6]. In October, 2011 the ACIP revised their recommendation for the HPV vaccine in males from permissive to routine [7]; however, it is still too early to determine the effect this new recommendation has had on vaccine uptake in the adolescent male population. Internationally, male HPV vaccination has also lagged, but Australia intends to begin routine school-based vaccination for males in late 2012 [8]. In order to understand more about HPV vaccination in males and to inform vaccine uptake interventions, the purpose of this study was to utilize qualitative methods to examine the decision-making process of parent-son dyads when deciding whether or not to get vaccinated against HPV.

Current literature on HPV vaccination in males predominantly focuses on the adult male population and has indicated high levels of acceptability of the quadrivalent HPV vaccine [9-13], but low awareness that the vaccine is currently available to males [11,14]. The few studies that have looked at the parents of adolescent males have found moderate [14] to high levels of vaccine acceptability with lower levels of intent to vaccinate their own sons [15]. Additionally, there were low levels of HPV knowledge [14] and limited awareness that the vaccine was available in males [14,16]. Furthermore, a survey of a national sample of parents of adolescent males found that only 2% of the sons had been vaccinated with 50% of these primarily due to doctor recommendation. At the same time, only 3% of the unvaccinated sons in this sample received a recommendation from their doctor [16]. Other research on providers’ attitudes towards male HPV vaccination have found that only 12% of pediatric and family medicine providers offered vaccination [17]. A recent study of older adolescents in England suggested that 16–18 year old males found the vaccine acceptable, although there has been no recommendation to vaccinate males in that country [18]. A more detailed recent review of male HPV vaccine literature can be found elsewhere [19].

While there has been more research on HPV vaccine in females, the subset of that literature that is most relevant to the current study is that of parent-daughter decision-making. This body of literature has shown that decision-making between parents and daughters is dynamic and involves a transactional process between dyad members [20,21]. Factors that influence a dyad’s decision to receive the HPV vaccine include physician recommendation [22-24], endorsement by significant others, such as parents, partners, and friends [21,25-27], and concern over the short length of time the vaccine had been licensed [28]. While we can utilize this information to inform the current study, current literature lacks information on how parents and sons make the decision to get vaccinated against HPV.

Due to the relatively recent licensure of the HPV vaccine in males in conjunction with the lack of knowledge about HPV vaccine decision-making in this population, we conducted a qualitative study of parent-son dyads immediately after they have been offered and made the decision to receive/refuse the HPV vaccine. Qualitative methods are ideal when little is known about a topic, such as point of care and joint decision-making. The objective of this study was to learn more about the structure and process of decision-making used by parents and sons when deciding to receive/refuse the HPV vaccine by examining, first, the initial reaction of each dyad member to the physician recommendation of the HPV vaccine, and then, the dyadic decision-making process.

## Methods

### Sample

Adolescent males, 13–17 years of age, and their parents or guardians (referred to as parents) were recruited from adolescent primary care clinics serving primarily lower income families in Indianapolis, Indiana, U.S. This age range was chosen because parental consent in Indiana is required for all vaccines for individuals under 18 years of age. Participants could not have received any HPV vaccine prior to the current health care visit and had to be accompanied by a parent or guardian. Otherwise eligible participants were excluded if a sibling already participated in the study. Both male and female parents were eligible to participate. A total of 23 parent-son dyads were approached for participation in the study. Two dyads refused participation, both due to time constraints. Therefore, a total of 21 dyads (42 interviews) participated in this study. This study was approved by the Indiana University Institutional Review Board.

All providers in these clinics routinely recommended the HPV vaccine to their eligible male patients, even prior to the revised ACIP recommendation. Lack of provider recommendation is a known barrier to vaccination [22], therefore, this design allows us to control for provider recommendation, and focus, instead, on the parent-son decision-making process.

### Procedures

During the clinic visit, prospective participants were seen by their provider and offered any ACIP-indicated vaccines as part of routine clinical care, including HPV vaccine. All study interviews occurred after the provider had finished and the parent-son dyad had either agreed or refused HPV vaccination. The research team approached the dyad immediately after the clinic visit, explained the study, and obtained parental consent and adolescent assent to participation. The parent-son dyad was then separated for simultaneous 30–60 minute interviews conducted in separate rooms with separate interviewers.

### Data collection

Once in the interview room, each participant was administered a demographic questionnaire by the interviewer to gather information such as age, marital status, education level, siblings/other children, and home composition. Subsequently, parents and sons completed a semi-structured interview aimed at eliciting the details of the decision-making process related to HPV vaccination. Interviewees were asked to reflect on the HPV vaccination decision just made. A total of 11 primary questions were asked of both parents and adolescents. Topics covered included HPV vaccine knowledge, information provided by the doctor, and the decision-making process. Each primary question was followed by probe questions. Interviewers listened for completeness and consistency in responses, asking participants to clarify and/or further explain their answers. An example of a primary question of parents with probes is illustrated by the following:

Primary Question: “How did you decide to get the vaccine?”

Probe 1: “What was said between you and your son when deciding to get the vaccine?”

Probe 2: “What factors played a major role in your decision?”

Probe 3: “What factors do you feel played a major role in your son’s decision?”

Each interview was audio recorded and transcribed. After each interview, the interviewer wrote up detailed field notes intended to document information not captured on the audio recording including an assessment of interview flow, pertinent background information on the participant, the setting and tone of the interview, as well as a detailed summary of the interview highlighting key points around decision-making.

### Analysis

Data were analyzed according to the method of inductive content analysis [29]. This method of content analysis is utilized when little is known about a topic or the knowledge is fragmented. This process of analysis includes open coding, creating categories, and abstraction, and tends to move from very specific to more general in order to create a picture of the larger whole. This method of analysis was chosen to facilitate the identification of dyad types, how they interact as a whole, and how they differ individually.

Two researchers read and openly coded 12 randomly chosen interviews identifying emerging issues, concepts, and themes surrounding the decision-making process used by male adolescents and their parent(s). The researchers then came together and discussed their individual codes and created a list of preliminary codes. Example codes include previous vaccine awareness, pain, protection, genital warts, and health. These codes were entered into NVIVO 9 (QSR International, 2011).The researchers then read through and coded the parent and son interviews, separately, in order to understand how the individual decision was made. Next, the researchers read through and coded the interviews in dyads in order to understand how the decision was made together and how each dyad member influenced the other. Once all interviews were read through both separately and together, a preliminary model was created. The model was expanded and adjusted throughout the process of coding, recoding, and sub-coding. The research team met numerous times until no new themes or changes to the model were detected. Disagreement between researchers was resolved through discussion.

## Results

### Participants

#### Sons

The majority of adolescents in this study were Black (n = 14), followed by Hispanic (n = 5), and White (n = 2). Adolescent age ranged from 13–17 with the majority being 14 years old (n = 6) followed by 15 and 16 (n = 5 each). All but two of the adolescents had siblings.

#### Parents

The majority of parents were female (n = 17). Parents ranged in age from 31–53 (M = 38.9, SD = 5.99). The majority of parents were single (n = 12), followed by married (n = 6). Half of the parents had at least a high school education (n = 11). The number of children of each parent ranged from 1–11 (M = 3.71, SD = 2.51).

### General description of the decision-making process

The conversation regarding vaccines was initiated at the end of the clinic visit by the physician for all but one dyad. In this dyad, the primary reason for the physician visit was vaccination for school, and thus, the topic was brought up by the parent. Unless the adolescent was up to date on all of their other vaccinations, the HPV vaccine was discussed along with the other routine vaccines given during adolescence. Most dyads could not recall exactly which vaccines were offered or received, for some this included HPV vaccine.

While the information presented to the dyads varied by physician (as recalled by each dyad member), each dyad felt the physician spent more time explaining the HPV vaccine than they did other vaccines offered that day. The reported physician message components included: (1) prevention against genital warts, (2) prevention against cancer, (3) recent approval in males, and (4) vaccine safety. While at least some dyads recalled the mention of cancer, the types of cancers varied. Most dyads recalled the discussion of anal and penile cancer. The dyads of one physician in particular tended to stress the rarity of these cancers. Additionally, a subset of physicians also reportedly mentioned protection against cervical cancer in girls. Although dyads could recall that most physicians mentioned that the vaccine was safe, most could not recall if the physician spoke specifically of the side effects of the vaccine, and most dyads did not ask about them. While providing information about the vaccine to the dyads, the physicians reportedly tended to look at both the parent and the adolescent throughout the conversation. This approach was not the case, however, for those adolescents with non-English speaking parents. In these instances, the dyads reported the physician looking at and discussing the vaccine directly with the adolescent.

After the vaccine was presented to each dyad by the physician, some asked the physician questions about the vaccine, including questions about vaccine safety, efficacy, and reasons for recent availability for males.

After questions about the vaccine were answered or the physician completed the discussion of the HPV vaccine (in instances where no questions were asked), the dyads were often asked if they wanted the HPV vaccine independent of their decision about the other vaccines offered that day. For the most part, the answer was given to the physician by the parent. However, in all but a few instances the adolescent also gave verbal or non-verbal cues of acceptance, which were often elicited by either the parent or the physician. When elicited by the physician, the adolescent was explicitly asked if they wanted the vaccine after the parent had given their response. Elicitation by parent occurred via a wide range of actions. Some parents explicitly asked the adolescent if they wanted the vaccination, similar to the physician. Other parents simply looked at their child, which then resulted in a verbal, “Okay,” or a nod, smile, or other method of non-verbal communication. While the physician elicitation occurred after the parental response, the parental elicitation took place either before or after the parental response was given. When adolescent opinion was not elicited by either parent or physician, it was due to the adolescent’s lack of engagement in the conversation or the parent’s strong opinion about the vaccine.

All but two of the dyads received HPV vaccine and most dyads were concordant in this decision. However, both the recall of the conversation with the physician and the reasons for vaccination often differed between parent and son (Table 1). For the two cases of vaccine refusal, one involved a father who did not want to make a decision without consulting his wife and the other one involved an adolescent son who was very scared of needles.

**Table 1:** Factors in individual HPV vaccination decision

**Factors of Individual Decision**	**Son**	**Parent**
Details from doctor	Protection	Genital warts
	Genital warts	Cancer
	Sexually Transmitted Infection	Sexually Transmitted Infection
	Cancer	Male availability
	Gonorrhea	Optional
	Male availability	Before sexual initiation
		Three shots
*Example Quote*	**Q1**.**1 **“*She was just saying it was for*, *you get it to prevent*…*you from catching genital warts and uh*, *she said it used to be for girls but uh*, *they just passed a law that it*’*s for boys and girls now *(*15 years old*).”	**Q1**.**5** “*She just mentioned the* [*vaccine*], *and I told her I didn*’*t realize it was for males also* [*be*]*cause my daughter just finished her series of it*. *And then she explained to me how it can help with things like genital warts and penile cancer*, *and just that there are things that can be prevented in boys the same as girls*. (*Female*, *40*).”
Reasons for vaccination	Protection	General protection of son
	Safety	Vaccine is safe
	Stay healthy	Sexual initiation soon
	Anticipated regret	
*Example Quote*	**Q1**.**2 **“*So I can stay healthy*. *I don*’*t want to mess with them *[*viruses*]. *I don*’*t want to die*, *or I don*'*t want to be sitting in a hospital*. *If there*’*s anything to keep myself away from it I*’*ma do it *(*15 years old*).”	**Q1**.**6** “*I try very hard to protect him as much as I can because I*’*m not there 24 hours a day*. *So*, *I can*’*t walk around with*, *I can*’*t put him in a condom and hold him in there* (*Female*, *33 years old*).”
Risks	Pain	Side effects
	Shot in penis	Questions about sex from son
*Example Quote*	**Q1**.**3**”*It hurts*. *I believe that that painful needle will protect me*, *so I*’*m gonna get it* (*13 years old*).”	**Q1**.**7 **“*We heard when she got hers*, *that some kid died because they got vaccinated*, *that in some places they have some negative reactions*, *but it*’*s a risk*. *Everything has a risk *(*Female*, *38 years old*).”
Benefits	Protection	Genital warts
	Safety	Cancer
	Stay healthy	Sexually Transmitted Infection
*Example Quote*	**Q1**.**4**”*Like this keeps you safe so you won*’*t be like hurt or anything like that*. *I guess just keep you healthy*, *make sure you like don*’*t spread it or anything like that *(*15 years old*).”	**Q1**.**8 **“*Well*, *the good thing is that if he decides to get sexually active that he*’*s protected on that* [*HPV*]. *Along with all the other protections that are out there *(*Female*, *34 years old*).”

### HPV vaccine decision-making among parents

Parents recalled a wide variety of information from the conversation with providers. The most common piece of information recalled was that the vaccine protected against genital warts, followed by cancer, and an STI. Other pieces of information commonly recalled by parents included the recent vaccine availability for males, that the vaccine was optional, and that the vaccine should, ideally, be given before the adolescent becomes sexually active. Most did not mention that HPV vaccination involves three doses.

The most commonly stated reason for getting sons vaccinated was parental interest in protecting their children from harm. Parents felt it was their job to protect their sons, and as long as the vaccine was safe, they felt that their sons should get it. Some parents specifically mentioned prevention of genital warts and/or cancer, but the large majority discussed protection and safety in more general terms. One parent felt the vaccine’s ability to prevent her son from spreading the infection was a benefit. Many parents were concerned that adolescents in general are having sex at younger ages, and that there are more STIs present than when they were growing up. For example, one parent said, “Cause…kids now a days are just too active. I mean, there’s just too much out there. It ain’t like it was when we was coming up (Female, 34 years old).” An added worry was parental concern about limited ability to monitor their sons’ activities (see Table 1, Q1.6 for example quote). Even for parents who were confident that their sons were not having sex, an important reason for vaccination was the realization that their sons may soon become sexually active.

With respect to safety, the large majority of parents felt there were no significant risks associated with HPV vaccination. Only two of these parents recalled the physician discussing potential minor side effects associated with the vaccine (e.g., pain at injection site) and they did not view these side-effects as reason to not vaccinate. Two parents felt that there were potential risks associated with the vaccine (e.g., worries that the adolescent will incorrectly assume that the parent endorses initiation of sexual activity). Nonetheless, in both of these cases the sons received HPV vaccine.

### HPV vaccine decision-making among sons

The majority of adolescents recalled the physician mentioning that the vaccine was preventive and they most frequently mentioned protection against genital warts, followed by protection against an STI and cancer. However, a number of adolescents could not recall anything the physician said about the HPV vaccine, and a small number thought the vaccine protected against gonorrhea. Reasons for choosing to get vaccinated included protection against disease, to stay healthy, and feeling it would be their fault if they did not get the shot and ended up catching the disease (i.e., anticipated regret).

Examination of individual decision-making processes showed that the sons actively weighed their perceived risks and benefits of HPV vaccination. Most of the adolescents who said that they wanted the vaccine talked about the benefits, but reported few perceived risks. When perceived risks were present, pain related to the injection was the most common concern. However, in these cases, the adolescents stated that the pain was not enough of a reason to refuse vaccination (see Table 1, Q1.3 for example quote). Some of the adolescents who initially were reluctant to get vaccinated were concerned that they were going to get the shot in their penis. After it was clarified that the shot would be in their arm, they went ahead and got vaccinated.

### Dynamics of parent-son decision-making process

Although many dyads were in agreement in their initial views toward vaccination, there was some disagreement. For the most part, the disagreement was due to the parent endorsing vaccination, and the son expressing reluctance. The most common reasons given for the son initially not wanting the vaccine were dislike of needles, being abstinent, and not understanding why the vaccine was needed. When one member of the dyad was undecided and the other member held a decisive opinion, the final decision coincided with the wishes of the latter.

**Figure 1:**
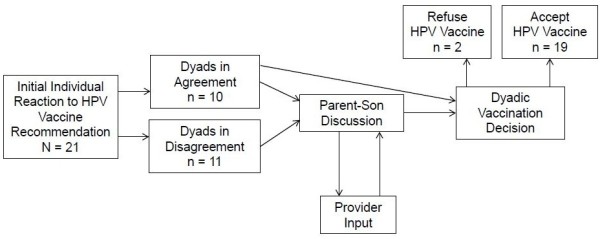
**HPV vaccine decision**-**making process as reported in the interviews.**

Disagreement was resolved via a conversation between parent and son, with some dyads bringing the physician into the conversation. For example, in one dyad it was determined that the reason the son did not want to get vaccinated was because he did not understand why he needed the vaccine as he was not sexually active, and did not plan on becoming active in the near future. In this case, physician input was elicited by the parent to explain why the vaccination was needed now. Ultimately, there was agreement across all dyads regarding the final vaccination decision, with the majority of dyads accepting vaccination (Figure 1).

Analysis of parent-son dyads showed that each member of the dyad had one of three possible views regarding the balance of decision-making. These included the perception of the parent as the decision-maker, the son as the decision-maker, or the parent and son both participating in the decision. Example quotes from dyads in each category are presented in Table 2. In about half of the dyads, there was concordance regarding decision-making, with most of these dyads perceiving the decision as either shared or made by the son. Even when there was discordance, in most cases, parents and sons ultimately agreed on the final vaccination decision.

**Table 2:** Criteria for role assignment in dyadic decision to get vaccinated

	**Parent**	**Both**	**Son**
**Criteria**	·Parent and/or son explicitly stated parent made decision on own.	·Son asked questions and/or was engaged in the conversation, and the parent stated their opinion or gave an answer to the doctor.	·Parent explicitly stated that they let their son make the decision.
	·Parent gave the answer to the physician without any attempt to include their son in the decision.	·Parent explicitly asked for son’s opinion	·Son explicitly stated that he made the decision alone.
***Example Quote***	**Q2**.**1***I looked at him and he looked at me and I said*, “*You*’*re gonna get that*.” *I mean*, *it wasn*’*t even a question*. *He*’*s 15 years old* (*Female*, *35 years old*).	**Q2**.**2***My mom looked at me and asked me if I wanted it and I said yea*. *Then my mom said yea *(*Son*, *15 years old*).	**Q2**.**3***Interviewer*: *Who do you think made the decision*?
			*Son*: *Me*.
			*Interviewer*: *So*, *what kinds of things were going through your head when you decided that*?
			*Son*: *I don*’*t want to get no shot right now* (*Son*, *16 years old*).

The majority of sons felt that they had a role in the vaccination decision, and most of their parents agreed. Most adolescents expressed positive feelings about being included in this decision and many said that this was the first time they had experienced any level of autonomy when it came to decisions about their health. For example, when asked how being included in this decision made him feel, one participant responded: “This was like one of the first things that I really got the decision on. And I kinda made the decision wisely because anything to make me stay healthy I’d rather do that (Son, 13 years old).” Many parents also felt this was the first time their son was included in a medical decision and viewed the issue as a matter of personal choice. For example, when asked if adolescents should be forced to get this vaccine, one parent responded, “I mean because, it’s like sex is their own, that’s up to them to make that decision. It’s their sexual decision, so as far as forcing them, it’s like forcing them to carry a condom in their pocket (Male, 31 years old).”

When both dyad members endorsed shared decision-making (n = 5) parents also reported open communication about sex or awareness that their sons were sexually active. When both dyad members agreed that the son made the decision, parents typically indicated that they did not openly communicate about sex and were unsure of their child’s sexual activity or confident that their child was not having sex.

## Discussion

The findings from this study suggest, in many cases, HPV vaccine decision-making for adolescent males involves an interactive process between parent and son, with important contributions from the health care provider. We found that protection against genital warts was perceived as a major benefit of vaccination, followed by cancer prevention. Although each of the providers reportedly discussed the HPV vaccine protecting against both genital warts and cancer, both parents and sons emphasized protection against genital warts as a main reason for vaccination. This is different from what has been found in the female population [30], but consistent with past research from mothers of sons [16,31], and should be considered by clinicians when recommending the vaccine to adolescent males and their parents.

When elaborating on protection, parents tended to focus on the need to protect their child from harm anyway they could. Complementary to this, sons focused on the need to maintain their health. This finding is consistent with other research on vaccine acceptability [32] and suggests that the way clinicians present the vaccine may need to address vaccination as a health maintenance as well as a disease prevention measure.

Although this study took place in adolescent-focused clinics with experienced adolescent health care providers, parents and sons still came away with some notable misinterpretations about both the benefits and risks of vaccination. Quite a few sons were concerned about getting an injection in their penis. Additionally, some parents and sons thought the HPV vaccine would protect them against other STIs including herpes, gonorrhea, and HIV. These misinterpretations emphasize the importance of clinicians providing clear and repeated information when presenting the HPV vaccine to adolescent males and their parents.

While the details of the conversation with the doctor differed between dyad members, the general components of the message were similar. However, there were still quite a few dyads that were in disagreement regarding their initial reaction to the vaccine. Through verbal and non-verbal communication, as well as periodic information provision by the clinician, disagreements were resolved and dyads came to final vaccination decisions together. The involvement of sons in the decision-making process was perceived to be a unique and positive experience by both parents and sons, which is consistent with other research on HPV vaccination [14,15], though past research on hypothetical STI and HPV vaccines found mixed results on autonomy and locus of decision-making [28,33-35]. Nonetheless, the increased participation in vaccination decision-making that we identified should be considered by clinicians as their patients progress into adolescence.

### Limitations

While the qualitative design of this study allowed us to examine in-depth information about parent-son decision-making around the HPV vaccine, there are a number of limitations. Although a sample size of 21 pairs of interviews is adequate for qualitative research, it limits the generalizability of these findings. Thus, care should be taken when applying the findings of this study to a larger population. Additionally, while this study provides a good starting point to understanding the process of vaccine decision-making among parents and sons, the data collection sites were clinics with particularly high adolescent vaccination rates. Research should be conducted in clinics with low vaccination rates as well as with non-clinical samples in order to better understand the range of issues surrounding HPV vaccination in males. Furthermore, although the study population included males ages 13–17, the vaccine is licensed to males ages 9–26. Therefore, care should be taken when applying these results to the entire range of eligible males. Finally, it is important to acknowledge the inherent power differential between parents and their sons, particularly in the context of a health care visit. It is possible that this power differential may have influenced, to some degree, the way sons participated in the decision-making process. We made efforts to minimize this issue by interviewing parents and sons separately. Future studies will want to explore, as well, the ways in which vaccination status of sisters may influence parent-son decision-making about HPV vaccination.

## Conclusions

There were many perceived benefits and few perceived risks associated with HPV vaccination of males. Decision-making about vaccination was an activeprocess involving both parents and sons. The health care provider was seen as playing an essential role in providing information and correcting misperceptions about HPV vaccination. Furthermore, this particular decision may provide an opportunity for the adolescents to become involved with decisions about their health care. Clinicians may use the results of this study to inform the way they present information on HPV vaccine to adolescent male patients and their parents.

## Abbreviations

ACIP: Advisory committee on immunization practices; HIV: Human immunodeficiency virus; HPV: Human papillomavirus; STI: Sexually transmitted infection.

## Competing interests

GDZ has served as a paid research consultant to Sanofi Pasteur. ABA, NWS, and GDZ are investigators on investigator-initiated research studies funded by Merck & Co., Inc. including the grant that funded the research reported in this manuscript (MISP #38094). The funder did not have a role in the study design, in the collection, analysis, and interpretation of data, in the writing of the report, nor in the decision to submit the article for publication. All other authors declare that they have no competing interests.

## Authors’ contributions

ABA contributed to the conception and design of the study, participated in acquisition of the data, analysis, and interpretation of the data, and drafted the manuscript. NWS contributed to the conception and design of the study, participated in acquisition of the data, analysis, and interpretation of the data, and critically revised the manuscript for important intellectual content. MAO, DH, and MR contributed to the design of the study, and critically revised the manuscript for important intellectual content. GDZ contributed to the conception and design of the study, interpretation of the data, and critically revised the manuscript for important intellectual content. All authors read and approved the final manuscript.

## Pre-publication history

The pre-publication history for this paper can be accessed here:

http://www.biomedcentral.com/1471-2431/12/192/prepub
